# Assessing the Efficacy of Delivering Hybrid Dementia Care in a Resource Scarce Rural Community

**DOI:** 10.1093/geroni/igaf122.4069

**Published:** 2025-12-31

**Authors:** Vi Belanger, Chris Beaulieu, Lenard Kaye, Rachel Coleman

**Affiliations:** Aroostook Area Agency on Aging, Presque Isle, Maine, United States; Aroostook Area Agency on Aging, Presque Isle, Me 04769, Maine, United States; University of Maine, orono, Maine, United States; University of Maine Center on Aging, Orono, Maine, United States

## Abstract

Older adult cognitive health services, especially dementia care, has been an especially scarce resource in rural communities. The Aroostook Memory Care Center (AMCC), in northern Maine, is testing the efficacy of delivering a hybrid combination of on-site and remote dementia care based at an Area Agency of Aging. AMCC is located in Aroostook County, the largest county east of the Mississippi River (6,700 sq. mi.) with all 24 census tracts designated rural, and the closest dementia specialty practice 150+ miles away. A 3-year ACL grant has enabled delivery of services for persons with dementia (PWD), support for caregivers, and community education. A process protocol for referrals and intake, care coordination and planning, interdisciplinary case reviews, remote specialty geriatrician neuropsychology assessments, and connections to AAA and other community services has now been established and successfully implemented. A Memory Center flow pathway protocol has been constructed and has been adhered to over the life of the program. Over a 15-month period, 282 referrals, 51 on-line referrals to specialty dementia care were made and 206 PWDs and 149 caregivers received services. Pre- and post-measures of the result of on-site and remote training and clinical interventions (changes in levels of understanding Alzheimer’s and dementia; levels of patient and caregiver preparedness because of expert consultation; and increases in caregiver quality of life) confirm significant participant gains. Strategies for sustaining a hybrid dementia care initiative in rural communities are offered focused on creating operational efficiencies, developing a reimbursement model, and tapping sources of philanthropy.

